# Prevalence of Tobacco Use in Young Adult Literate Girls of 18-25 Years in Meghalaya, India: A Cross-Sectional Study

**DOI:** 10.31557/APJCP.2021.22.9.2923

**Published:** 2021-09

**Authors:** Sutapa Biswas, Judita Syiemlieh, Roken Nongrum, Shashi Sharma, Maqsood Siddiqi

**Affiliations:** 1 *Cancer Foundation of India, Kolkata, West Bengal, India. *; 2 *Civil Hospital, Shillong, Meghalaya, India. *; 3 *National Institute of Cancer Prevention and Research, NOIDA, U.P, India. *

**Keywords:** North-eastern India, tobacco, alcohol, young girls, students, education, family income

## Abstract

**Background:**

Several national-level surveys have recognized a high prevalence of tobacco use in North-eastern (NE) India. However, information on tobacco use in specific population subgroups still lacks from the region. The present study determines the prevalence and influence of determinants like education and family income on tobacco use in senior school and college-going girls in Shillong, Meghalaya. Additionally, the prevalence of dual use of smoking and smokeless (SLT) tobacco with alcohol and non-tobacco Pan Masala has been examined.

**Methods:**

A cross-sectional study was conducted among 18-25 years girls in educational institutions in Shillong. Data were analysed using statistical software SPSS version 22. The categorical data presented as frequency (%). Chi-square was employed to see the association between variables.

**Results:**

(i) 8.10% of girls were current users of all forms of tobacco; 1.85% were smokers, 4.0% used SLT, and 2.25% were dual users of tobacco (ii) Of the 6.25% current users of SLT, 5.4% used a single smokeless tobacco product whereas 0.85% used multiple products of SLT (iii) 79.5% of tobacco smokers and 30.2% of current SLT users were dual users with alcohol (iv) 37.2% tobacco smokers and 18.5% SLT users were dual users with non-tobacco Pan Masala (v) Tobacco smoking was positively associated with educational status and family income whereas SLT use was independent of educational status and income of girls’ families.

**Conclusions:**

The study shows a relatively high prevalence of tobacco use and dual tobacco use with alcohol in the study population of educated young adult girls, underscoring a public health concern. It is recommended that an education-based comprehensive awareness program be initiated for tobacco and alcohol control in Meghalaya to improve knowledge and health-seeking behaviour change in this high- risk subgroup to control increasing NCDs.

## Introduction

Tobacco use is an established risk of cancer of at least eight different anatomical sites and several other non-communicable diseases (NCDs) such as cardiovascular disease and stroke (Thakur et al., 2011; Gupta et al., 2019; Shrestha et al., 2019). Nearly 8 million deaths globally and about one million deaths each year in India are attributed to tobacco (Fact Sheet India, 2018; WHO global report, 2019). According to the population-based cancer registry (PBCR), 27.1% of India’s cancer cases are tobacco-related (Report of NCRP, 2020). Several national surveys (NFHS-4, 2016; GATS-2, 2018) and geospatial distribution studies have demonstrated a high prevalence of tobacco use in North-eastern (NE) India (Fu et al., 2014, Krishnamoorthy and Ganesh, 2020). With 66.9% tobacco-related cancers in men and 43.1% in women, Meghalaya in NE has the maximum occurrence of tobacco-related cancers in the country (Report of NCRP, 2020).

Meghalaya also shows a high prevalence of tobacco use with 47.0% prevalence in adults (15 years and above), having 26.7% smokers, 15.4% users of smokeless tobacco (SLT), and 4.9% of dual users of tobacco, which is higher than the national average of 28.6%, 7.2%, 17.9%, and 3.4% respectively (GATS-2, 2018). In addition, the prevalence of any form of tobacco use in Meghalaya’s women is 34.2%, with 9.5% smokers and 29.0% SLT use which is also nearly 2.5 folds higher than the national average of 14.2%, 2.0%, and 12.8%, respectively (Ladusingh et al., 2017; GATS-2 Meghalaya Fact Sheet, 2018). 

Implementing anti-tobacco laws followed by effective public health initiatives has resulted in a modest to marked decline in the overall consumption of tobacco in most states in India (Abdulkader et al., 2019). Nevertheless, more specific data on the prevalence of tobacco usage in different socio-economic, occupational, and educational groups and subgroups are required in different states to develop targeted public health interventions. A crucial subgroup where essential data is deficient is that of young boys and girls. Understanding the trend of youth’s tobacco use is considered vital for assessing the prospective risk of various NCDs in a population (Viner et al., 2017). It also helps formulate effective public health measures to control the epidemical spread of tobacco use in the youth. 

 In the present study, we report the first data on tobacco smoking, smokeless tobacco (SLT), and dual tobacco use in senior school and college-going girls of 18-25 years in Shillong, Meghalaya. The study evaluates the dual use of tobacco smoking and SLT with alcohol and non-tobacco Pan Masala. The influence of girls’ current educational status and family income on the prevalence of tobacco smoking and SLT is also examined.

## Materials and Methods

*Study setting and Design*

Details of the study area and broad demographic details of the cohort of girls from randomly chosen educational institutions are already described earlier (Biswas et al., 2020). Briefly, an educational institution-based cross-sectional survey on tobacco use was conducted among senior school and college girls of 18-25 years in urban and peri-urban areas in Shillong (Meghalaya), India. The survey conducted between June 2015 and December 2016 included 1904 girls using a pretested self-administered questionnaire in English. The study was conducted along with the survey on breast cancer awareness. Therefore, in addition to questions relevant to cancer awareness and risk factors of breast cancer, several questions on the use of tobacco, alcohol, and non-tobacco Pan Masala (PM) were included in the questionnaire. We have used the 18-25 years age group to have a broader educational level and have girls with knowledge of at least high school level. The chosen age group also had the advantage of exemption from parental consent to introduce the self-administered questionnaire among girls below 18.

*Statistical Analysis*

The data were analysed using statistical software SPSS (IBM Corp Ltd. Armonk NY, USA) version 22. The categorical data presented as frequency (%) and the comparison was made using Chi-square or Fisher exact test. In addition, Chi-square and test of single proportion were employed to see the association between several variables.

## Results

*Demographic Characteristics*

Age: Out of the 1904 girls, 1629 (85.5%) were 18-21 years and 275 (14.5%) between 22 and 25, with an average age of 19.9 ± SD1.5 years. 

*Education*

Three hundred and nine girls (16.2%) were from the final year at school, 1127 (59.2%) girls were doing bachelor’s courses in arts and social sciences, and 396 (20.8 %) were from the science stream. Seventy-two girls (3.8%) were pursuing post-graduate and higher education. 

*Family Income*

The family income of 408 girls (21.3 %) was less than Indian Rupees (INR) 5,000 per month. Seven hundred and ninety-eight girls (42.0 %) came from families whose income was between INR 5001 and INR15,000, 394 (20.7 %) were from families with a monthly income between INR 15,001 and 25,000, and 201 (10.5 %) girls from families with an income between INR 25,001 to INR 50,000. One hundred and three girls (5.4%) belonged to higher-income families, i.e., INR 50,001 and above per month. 

*Prevalence of Smoking Tobacco and Smokeless Tobacco (SLT)*

As shown in Table 1, 95 (5.0%) respondents were ever smokers of tobacco (cigarettes), with 78 (4.1%) of them being current smokers and 17 (0.9%) girls stated that they had smoked in the past. Among the current smokers, most of the girls were occasional smokers (57; 73.1%), and only 21 girls (26.9%) smoked regularly (p<0.001). It is evident from the dual usage data that 43 girls were also users of smokeless tobacco (SLT), showing that 55.1% of the current tobacco smokers also used SLT concurrently. It also shows that 2.25% of the study sample were users of both forms of tobacco. 

The data on smokeless tobacco (SLT) show that 119 girls (6.25%) of the study cohort were current users of SLT (Table 2). Of these103 (5.4%) girls used a single smokeless tobacco product, whereas 16 girls (0.7%) used multiple products of SLT. Of the single SLT product users, while most preferred was gutka (72;69.9%), 15.5% (16) were using zarda, and 14.5% (15) used other forms of SLT (p<0.001). At the same time, among the multi SLT users, nine girls used gutka and zarda, and seven girls use gutka with other forms of SLT. The dual usage data of both forms of tobacco given in Table 2 shows 43 girls also used smoking tobacco smoking, implying 36.1% of current SLT users smoked tobacco or 2.25% of the study population were users of both forms of tobacco (p<0.001). Thus, statistical significance was observed between habit and users as indicated.

The data given in Table 1 and 2, therefore, show that 8.10% of girls in the survey sample were current users of any form of tobacco; 1.85% used only smoking tobacco; 4.0% of girls used SLT, and 2.25% were dual users of smoking and smokeless tobacco. 

The difference in the prevalence of current tobacco use, both smoking and SLT, in the age groups of 18-21 years and 22-25 years was not found statistically significant.

*Dual users of Tobacco with Alcohol and Pan Masala*

Smoking Tobacco: As given in Table 1, 177 girls, or 9.3% of the total sample, were current users of alcohol (part data from Biswas et al., 2020). Of the 78 current tobacco smokers, 62 girls were also currently taking alcohol, implying that 79.5% of current tobacco smokers also consumed alcohol, or 3.25% of the study population were alcohol drinkers along with smoking tobacco.

Similarly, the dual use of tobacco with non-tobacco Pan Masala (PM), a popular mouth freshener among youth in South Asian countries, including India, was also studied. It is mentioned in Table 1 that out of 1904 girls in the survey, 183 girls, or 9.6% of the total sample, were current users of PM (data not shown separately). Of the current smoking tobacco users, 29 girls, or 37.2%, were also consuming non-tobacco Pan Masala, or 1.5% of the study cohort were dual users of smoking tobacco and PM.

Smokeless Tobacco (SLT): Table 2 shows the data on dual use of SLT with alcohol and non-tobacco Pan Masala (PM). Thirty-six (36) girls used alcohol with SLT, meaning 30.2% of the current users of SLT or 1.9% of the study cohort was a dual user of SLT with alcohol. It is also shown in Table 2 that 22 SLT using girls used non-tobacco Pan Masala, meaning that 18.5% current users of SLT also used PM, or 1.15% of the study population was a dual user of SLT with PM. 

*Association of Tobacco Smoking and SLT with the level of Education and Family Income*

The association of girls’ current tobacco smoking habits with their educational levels and their family’s monthly income was studied using Chi-square statistics. As shown in Table 3, it is evident that the pattern of tobacco smoking in girls is positively associated with the educational status of girls [*χ*^2^ (9; N=1904) 25.898, p=0.002] as well as the monthly income [*χ*^2^ (12; N=1904) 36.550, p<0.001] of their family. 

Similar analysis on the relationship of current SLT users shows that the association of SLT use by the girls in the study sample is not a statistically significant either with the educational status of girls [*χ*^2^ (3; N=1904) 3.089, p=0.377] or with the monthly income of their family [*χ*^2^ (4; N=1904) 2.774, p=0.596].

**Table 1:** Prevalence of Tobacco Smoking and Duel Use with Smokeless Tobacco (SLT), Alcohol and Non-Tobacco Pan Masala

Use of Tobacco	Total Sample	Current Users		Total Current user	Had in the past	Ever Users	Dual Use with* n (% of Current Smokers)			p value
	n	Regular n (%)	Occasional n (%)	n (%)	n (%)	n (%)	Smokeless Tobacco	Alcohol	Pan Masala (Non-Tobacco)	
Tobacco Smoking	1904	21 (1.1)	57 (3.0)	78 (4.1)	17 (0.9)	95 (5.0)	43 (55.1)	62 (79.5)	29 (37.2)	P<0.001

*Current Smokeless Tobacco Users, 119/1904 (6.25%); Alcohol Users, 177 / 1904 (9.3%); Pan Masala Users, 183 /1904 (9.6%); Statistical association was analysed between habit and users by Chi-square statistics

**Table 2:** Prevalence of Smokeless Tobacco (SLT) and Duel Use with Smoking Tobacco, Alcohol and Non-Tobacco Pan Masala

Use of Tobacco	Total Sample	Single SLT Users	Multi SLT Users	Total SLT Users	Dual Use with** n (% of Current SLT users)			p value
	n	n (%)	n (%)	n (%)	Smoking Tobacco	Alcohol	Pan Masala Non-Tobacco	
Gutka	1,904	72 (3.8)	16 (0.85)	88 (4.65)	43 (36.1)	36 (30.2)	22 (18.5)	P<0.001
Zarda	1,904	16 (0.8)	9 (0.48)	25 (1.30)				
Other forms of SLT	1,904	15 (0.8)	7 (0.35)	22 (1.15)				
Total		103 (5.4)	16* (0.85)	119 (6.25)				

*9 Gutka/Zarda; 7 Gutka/other SLTs; ** Current Smoking Tobacco Users, 78/1904 (4.1%); Alcohol Users, 177 / 1904 (9.3%); Pan Masala Users, 183 /1904 (9.6%); Statistical association was analysed between habit and users by Chi-square statistics

**Table 3:** Association of the Use of Smoking and Smokeless Tobacco with Educational level and Family Income

Education /Family Income	Total Sample	Current use of Smoking Tobacco	Current use of Smokeless Tobacco
	n	n (%)	n (%)
Girls’ Education level			
School	309	16 (5.2)	18 (5.8)
Graduation (Arts)	1,128	37 (3.3)	68 (6.0)
Graduation (Sci)	395	17 (4.3)	25 (6.3)
Post-Graduate	72	08 (11.1)	08 (11.1)
Chi-square	χ2 (9; N=1904) 25.898, p=0.002		χ2 (3; N=1904) 3.098, p=0.377
Girls’ Family Income			
< 5,000 INR*	408	6 (1.5)	23 (5.6)
5,001 - 15,000 INR	798	28 (3.5)	55 (6.9)
15,001 - 25,000 INR	394	18 (4.6)	19 (4.8)
25,000 - 50,000 INR	201	16 (7.9)	14 (6.9)
> 50,001 INR	103	10 (9.7)	08 (7.7)
Chi-square	χ2 (12; N=1904) 36.550, p<0.001		χ2 (4; N=1904) 2.774, p=0.596

*INR, Indian Rupees

## Discussion

Given the extensive evidence favouring a high tobacco consumption in North-eastern states in India and particularly in Meghalaya (NFHS-4, 2016; NFHS-5, 2020; Ladusingh, 2017; GATS-2, 2018; Sarkar et al., 2019), the present study endeavoured to determine the tobacco use among senior school and college-going girls of 18-25 years in the capital town, Shillong. Our results show that the prevalence of current users of any form of tobacco was 8.10%; 1.85% were tobacco smokers, 4.0% of girls used SLT, and 2.25% were dual tobacco users. Further, in the study population, 5.0% of girls were ever smokers, 4.1% were current smokers, and the rest had ceased smoking. On the other hand, of the 6.25% SLT users, 5.4% used a single smokeless tobacco product, and 0.85% used multiple SLT products. The dual usage data of tobacco showed that nearly one-third of SLT users (36.1%) were dual tobacco users, and little over half of the current smokers (55.9%) also used SLT (Table 1 and 2). The reported national figures (GATS-2, 2018) on girls in the near comparable age group of 15-24 years (any form of tobacco: 3.7%; smokers: 0.1%; SLT users: 3.6%) are relatively much lower than our data. The tobacco use in our study population is also much higher than those of 18-24 years old college girls in Mangalore; smoking tobacco, 2.1%, and SLT use, 3.3% (Jodalli and Panchmal, 2019). Likewise, the reported prevalence of any form of tobacco use in college girls from medical, nursing, engineering, arts and science faculties in Ernakulum, Kerala (Menon et al., 2020) being 1.0%, 0.5%, 1.2%, and 1.1%, respectively, are far lower than those found by us. The girls in our study also showed much higher tobacco prevalence than in female students from 25 Universities from low- and middle-income countries reported by Pelzer and Pengpid (2014). 

The girls in our survey sample consisted of the age group generally known to be vulnerable to the use of other intoxicants and addictive substances, besides tobacco (Pillai et al., 2014, Sharma et al., 2020). Therefore, the questionnaire included closed-ended questions on the use of alcohol and non-tobacco Pan Masala (PM). The choice of alcohol was evident for its proven adverse health effects and because its concurrent use with tobacco is well established (Fu et al., 2014). On the other hand, PM was included in the survey since its main ingredient, Areca nut is known for causing sub-mucous fibrosis and has a recognised causal link with oral cancer (Garg et al., 2015). 

The associated habit of tobacco and alcohol drinking, though long-established in India, shows wide geographical variation in the country (Gupta et al., 2005; Fu et al., 2014; Sunitha and Gururaj, 2014). While several reports show that the use of tobacco, alcohol and other substances of intoxication and addiction is quite common in North-eastern India (Chaturvedi et al., 2003; Hazarika et al., 2000; Mahanta et al., 2016), no comparative study on the prevalence of tobacco and alcohol is available on youth in Meghalaya. We found 79.5% of current tobacco-smoking girls also took alcohol, and 30.2% of current SLT users consumed alcohol. Although concurrent use of tobacco and alcohol is considered an established behavioural risk for NCDs (Sharma et al., 2020), fewer reports are available on its prevalence in youth in educational institutions, except for some studies on tobacco and alcohol use among medical students (Goel et al., 2015). 

The non-tobacco Pan Masala (PM) is used as a mouth freshener containing a mixture of betel nut, catechu, lime, and cardamom seeds with flavouring substances. The PM users display chromosome aberrations and sister chromatid exchange in the peripheral blood cells and micro-nucleated cells in the exfoliated buccal mucosa cells (Dave et al., 1991). Consequently, PM users are known to have high odds of developing multiple oral precancers like those induced by betel quid and areca nut and its products (Jacob et al., 2004; Ali et al., 2013; Garg et al., 2015). Our results show that 9.6% of the respondents were current PM users, of which 8.4% were using PM alone, the remaining were dual users with either form of tobacco. A study in Lucknow on 453,823 individuals found PM commonly used by boys and girls in the 15-19 years age group with a prevalence of 3.0%. At the same time, tobacco users were predominantly between the age of 25-35 years among males and 35-39 years among females (Mehrotra et al., 2017).

Having a broader range of educational status and known demographic profile of girls in the present study, we used the Pearson chi-square test (*χ*^2^) to assess the statistical significance of the association of educational level and monthly income of respondent’s family on the prevalence of the use of tobacco. As shown in Table 3, tobacco smoking was significantly associated with girls’ education and their families’ monthly income (p<0.006), indicating that girls with higher academic status have an increased propensity for tobacco smoking. Similarly, the family’s income was positively associated with tobacco smoking (p<0.001), implying that girls from affluent families are more prone to smoking tobacco. On the contrary, neither the educational attainment of girls nor their families’ income significantly affected smokeless tobacco use. Thus, it implies that smokeless tobacco use by girls is independent of the education of respondents and the economic status of their families. 

Many population surveys have indicated that higher socio-economic background and literacy are associated with lower tobacco consumption (Rani et al., 2003, Palipudi et al., 2014; Grover et al., 2020). Though poverty and low literacy may carry the burden of tobacco use in specific socio-economical settings, it cannot be generalized as such since social determinants may differ in different population groups/subgroups. Agarwal and co-workers in a large cross-sectional study in India, report that the protective effect of education and wealth works well as far as biri smoking and smokeless tobacco is concerned and not for cigarette smoking in some populations (Agrawal et al., 2013). Another study using the World Health Survey data demonstrated that while tobacco smoking in many countries was nearly 2.5 times more prevalent in poor men, in many countries rich women were more inclined to tobacco use (Hosseinpoor et al., 2012). A direct association of higher education with tobacco is evident in the study where a prevalence of 8.0% tobacco use was reported among undergraduate students compared to 14.5% in post-graduate medical students drawn from 8 medical colleges in India (Goel et al., 2015). Thus, our results seem to introduce an element of intricacy in the perceived relationship of education and wealth with tobacco usage and calls for further validation with large sample-sized state-specific studies. 

In conclusion, it is shown that the prevalence of smoking and smokeless tobacco use in senior school and college-going girls in Shillong was substantially higher than reported for comparable and near comparable populations. We found that little over half of the current smokers (55.9%) and nearly one-third users (36.1%) of smokeless tobacco (SLT) were dual tobacco users. A large majority of tobacco smokers (79.5%) also took alcohol. The prevalence of non-tobacco Pan Masala was high (9.6%), and 37.2% of tobacco smokers and 18.5% SLT users also took PM. We have demonstrated that tobacco smoking was significantly associated with girls’ education and family’s monthly income. In contrast, smokeless tobacco was independent of girls’ education and the economic status of their families. Since the girls in the study sample are in their formative years, our results indicate a prospective public health concern, particularly for NCDs. Therefore, it is recommended that the state’s existing tobacco and alcohol control programmes be re-modelled to include education-based communication initiatives on the adverse health effects of tobacco and alcohol to encourage behaviour change amongst school and college girls. It will indeed help in pre-empting a prospective surge in the occurrence of non-communicable diseases.

## Author Contribution Statement

The authors confirm contribution to the paper as follows: study conception and design MS, SB; data collection and supervision of study: JS, RN; analysis and interpretation of results: SS, MS; draft manuscript preparation: MS, SB, SS; All authors reviewed the results and approved the final version of manuscript.
